# Median Nerve Measurement and Steroid Injection for Carpal Tunnel Syndrome: A Case Report

**DOI:** 10.5811/cpcem.7194

**Published:** 2024-07-11

**Authors:** Gregory Oliva, Joseph McShannic, Yonghoon Lee, Michael Shalaby

**Affiliations:** * Mount Sinai Medical Center Miami Beach, Department of Emergency Medicine, Miami Beach, Florida; † Herbert Wertheim College of Medicine at Florida International University, Department of Emergency Medicine and Critical Care, Miami Beach, Florida

**Keywords:** *carpal tunnel syndrome*, *median nerve*, *ultrasound*, *steroid injection*, *case report*

## Abstract

**Introduction:**

Carpal tunnel syndrome is an entrapment neuropathy that affects 3% of adults in the United States. The current techniques used for diagnosis have limited specificity/sensitivity, and the techniques used for treatment have limited efficacy.

**Case Report:**

A 34-year-old female presented to the emergency department with two months of worsening painful paresthesias in her right thenar eminence. Ultrasound was performed showing a median nerve area of 20.4 square millimeters within the carpal tunnel. Median nerve block was performed within the carpal tunnel causing complete resolution of her pain.

**Conclusion:**

Emergency physicians skilled in point-of-care ultrasound and needle-guided procedures can diagnose and treat carpel tunnel syndrome.

CPC-EM Capsule What do we already know about this clinical entity?

*Carpal tunnel syndrome (CTS) is prevalent, affecting 3% of adults in the United States, but diagnosis and treatment methods are limited in efficacy.*

What makes this presentation of disease reportable?

*Median nerve block in the emergency department (ED) efficiently aids in diagnosis and relieves newly diagnosed CTS.*

What is the major learning point?

*Emergency physicians skilled in point-of-care ultrasound and needle-guided procedures can diagnose and treat CTS effectively.*

How might this improve emergency medicine practice?

*Early ED diagnosis allows for prompt intervention, potentially improving patient outcomes.*

## INTRODUCTION

Carpal tunnel syndrome (CTS) is a common entrapment neuropathy caused by compression of the median nerve as it travels through the wrist within the carpal tunnel. Carpal tunnel syndrome accounts for 90% of all entrapment neuropathies and affects 3% of adults in the United States.^1^ Carpal tunnel syndrome induces pain, numbness, and paresthesias along the palmar aspects of the first, second, third, and lateral portion of the fourth digit, as well as the thenar eminence. In advanced disease, symptoms progress to weakness of thumb abduction, decreased grip strength, and thenar atrophy.^1^ While the diagnosis is clinical and is supported by findings such as Tinel sign (percussion of proximal wrist causing paresthesias) and Phalen sign (holding wrists in 60 degrees of flexion eliciting symptoms), physical exam maneuvers have a low sensitivity and specificity.^2^ The gold standard for confirmation of the diagnosis is a nerve conduction study.^3^

Ultrasound (US) measurements of the median nerve can be used to assess symptomatic patients, revealing an enlarged nerve area in individuals with CTS.^4^ Numerous strategies have been employed to alleviate the symptoms of CTS. These include diuretics, pyridoxine, non-steroidal anti-inflammatory drugs, yoga, steroid injections, US therapy, and acupuncture.^5^ These techniques can decrease inflammation and provide some relief but do not always cause complete resolution of pain. Herein, we describe a patient with new-onset CTS whose treatment in the emergency department (ED) with combined local anesthetic and corticosteroid injection provided complete relief of symptoms. This is the first case report detailing the utilization of ultrasound-guided median nerve block in the ED setting.

## CASE REPORT

**Image 1.**
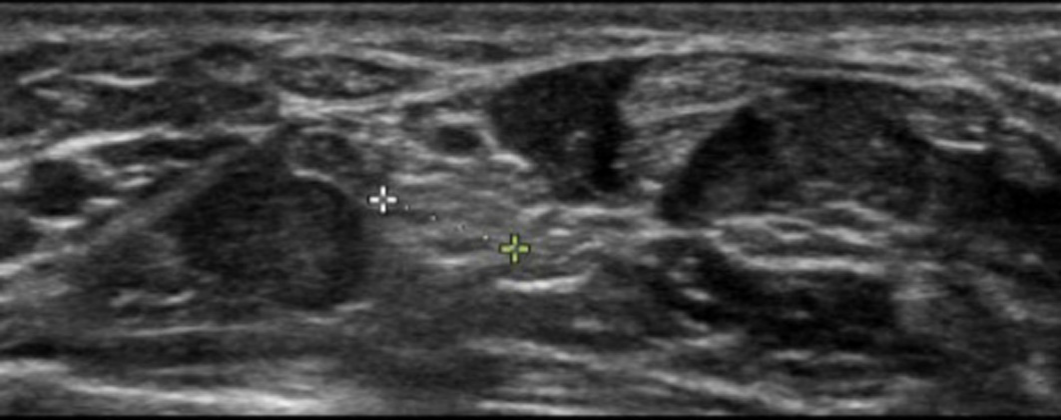
Enlarged diameter of the median nerve within the carpal tunnel, measuring 5.1 millimeters, which corresponds to an area of 20.4 square millimeters.

A 34-year-old female presented to the ED with two months of worsening painful paresthesias in her right thenar eminence. On physical exam, positive Tinel and Phalen signs were noted, along with reduced sensation of the palmar aspect of her first three digits. Muscular strength was 5/5 in bilateral upper extremities. The differential diagnoses encompassed cervical radiculopathy, thoracic outlet syndrome, pronator teres syndrome, and anterior interosseous neuropathy. Considering the symptom location and absence of sensory alterations, weakness, or tenderness proximal to the wrist, the leading explanation was compression of the median nerve within the carpal tunnel. Ultrasound was performed showing a median nerve area of 20.4 square millimeters (mm^2^) (reference range 8.5–10 mm^2^) within the carpal tunnel (Image 1).

The patient consented to a median nerve block, which was performed within the carpal tunnel with 10 milliliters (mL) of 0.25% bupivacaine and 1 mL of 40 milligrams (mg)/mL triamcinolone. After the procedure, the patient developed complete resolution of her pain. She was placed in a wrist splint and discharged home with information for hand surgery follow-up. Despite numerous phone calls, the patient was unfortunately lost to follow up.

## DISCUSSION

**Image 2.**
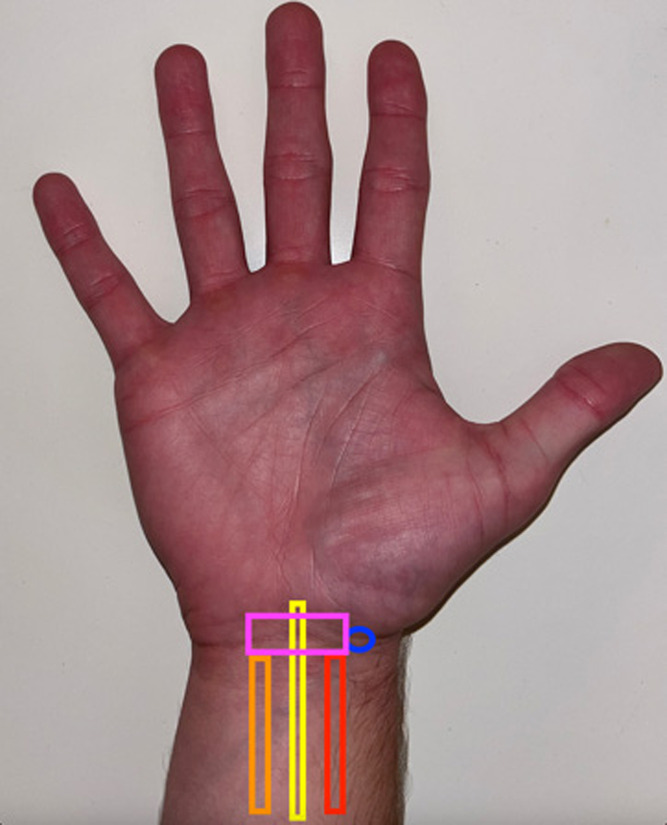
The external anatomy of the wrist for optimal ultrasound-guided needle placement is illustrated as follows: the blue circle denotes the needle insertion site, while the pink box represents the placement of the ultrasound probe. Additionally, the red, yellow, and orange boxes indicate the positions of the flexor carpi radialis, median nerve, and palmaris longus, respectively.

The median nerve arises from the anterolateral and anteromedial cords of the brachial plexus and comprises the sixth cervical to first thoracic nerve roots.^6^ Distally, the nerve courses deep to the flexor retinaculum and enters the carpal tunnel, traveling in an anterior and lateral direction alongside the tendons of the flexor digitorum superficialis.^6^ It is at this point that entrapment is most common. Beyond the carpal tunnel, it divides into a motor branch that serves the thenar compartment, as well as the first and second lumbricals, and a sensory branch that divides into four palmar branches for the fingers.^6^ To perform a median nerve block within the carpal tunnel, the nerve is first identified between the flexor digitorum superficialis and flexor digitorum profundus, and then traced distally to its position within the carpal tunnel.^7^ Once at this position, the nerve can be identified medial to the flexor carpi radialis and lateral to the palmaris longus (Image 2).

An in-plane approach is used to advance the needle before instilling local anesthetic around the nerve. Usually, ≤5 mL local anesthetic suffices for median nerve blockade. Local anesthetics such as bupivacaine and ropivacaine provide longer pain relief and are, therefore, preferred over lidocaine.^7^ While physicians in other specialties have previously employed this procedure, this case report represents the first documentation of ultrasound-guided median nerve block being used in an ED setting. In general, median nerve area of greater than 8.5–10 mm^2^ at the mid channel is associated with CTS.^4^ In our case, we calculated the median nerve area to be 20.4 mm^2^. With these findings, we deduced that the nerve was exhibiting signs of inflammation.

Carpal tunnel syndrome is a painful condition affecting >8 million people yearly.^8^ Carpal tunnel release is the second most common type of musculoskeletal surgery with over 230,000 cases annually.^8^ While the procedure can be effective at relieving pain, it is invasive and costly, and results are poorer for patients with longstanding disease.^9^ Patients with operative intervention earlier in the course of the disease have been shown to have better outcomes.^9^ To refer patients for treatment in a timely fashion, the diagnosis must be timely. While diagnosis is usually made clinically, calculation of the median nerve cross-sectional area and nerve blockade can provide a definitive diagnosis and timely analgesia. Furthermore, instilling corticosteroid within the sheath can provide prolonged analgesia. The introduction of dexamethasone has been observed to result in prolonged nerve block durations, reduced opioid requirements, and an extended time until the next analgesic dose is needed.^10^ ^–^ ^12^ When deciding whether corticosteroids should accompany local anesthetics for nerve blocks, the choice should hinge on the desired duration of the blockade and considerations of contraindications. According to the American Society of Regional Anesthesia and Pain Medicine, dexamethasone is among the frequently used agents for extending analgesia. However, official recommendations are impeded by the low quality and clinical diversity of published trials.^13^

Ultrasound guidance facilitates median nerve blockade by allowing emergency physicians to feasibly and precisely target the median nerve within the carpal tunnel. Therefore, instead of performing a median nerve block in the usual mid-forearm location, we opted to instill anesthetic around the nerve within the carpal tunnel to include a corticosteroid at the site of compression. Moreover, injection of anesthetic directly into the carpal tunnel has been shown to provide improved symptom severity and function.^14^

Nerve blocks may entail various complications, such as neuronal damage, bleeding, and local anesthetic systemic toxicity (LAST) syndrome.^15^ Peripheral nerve injury is an infrequent outcome, with an estimated incidence ranging from 0.5–1.0%.^15^ Although nerve injury encompasses a spectrum of issues, permanent nerve damage occurs in approximately 1.5 cases per 10,000.^15^ Inadvertent vascular puncture poses risks of bleeding with hematoma formation or, if the anesthetic enters the vessel, triggering LAST syndrome.^15^ Symptoms of LAST syndrome can range from mild, such as circumoral numbness, metallic taste, and auditory changes, to severe, including seizures, coma, respiratory arrest, hypotension, ventricular arrhythmias, and cardiac arrest.^15^ Recent studies suggest that using US guidance reduces the likelihood of inadvertent vascular puncture, thereby mitigating these complications.^15^

While dexamethasone is commonly employed to extend analgesia’s duration in peripheral nerve blocks, its usage is cautioned against in diabetic patients due to the associated hyperglycemic response.^16^ Absolute contraindications for corticosteroid injection include local infection, sepsis, and bacteremia, as they pose risks of infection dissemination. Relative contraindications include juxta-articular osteoporosis due to concerns about exacerbating bone density loss, coagulopathy, and injections three times annually or within a six-week period.^17^

## CONCLUSION

Median nerve block is an effective and efficient way to both diagnose and provide relief of newly diagnosed carpal tunnel syndrome that presents to the ED, and emergency physicians should consider using it within their practice for such.
